# Soluble Tim3 detection by time‐resolved fluorescence immunoassay and its application in membranous nephropathy

**DOI:** 10.1002/jcla.23248

**Published:** 2020-02-20

**Authors:** Ming Chen, Liang Wang, Yigang Wang, Xiumei Zhou, Xinyuan Liu, Hao Chen, Biao Huang, Zhigang Hu

**Affiliations:** ^1^ College of Life Sciences and Medicine Zhejiang Sci‐Tech University Hangzhou China; ^2^ Wuxi People's Hospital Affiliated to Nanjing Medical University Wuxi China; ^3^ Wuxi Children's Hospital Wuxi People's Hospital Affiliated to Nanjing Medical University Wuxi China

**Keywords:** membranous nephropathy, soluble Tim3, time‐resolved fluorescence immunoassay

## Abstract

**Background:**

We aimed to develop a time‐resolved fluorescence immunoassay (TRFIA) for detecting soluble T‐cell immunoglobulin and mucin domain 3 (sTim3) in serum samples and to demonstrate a preliminary application of this method in membranous nephropathy (MN).

**Methods:**

sTim3 TRFIA was developed, and the sTim3 concentration in the serum of patients with MN and healthy individuals was detected using a sandwich method.

**Results:**

The sensitivity of the developed sTim3 TRFIA was 0.66 ng/mL, higher than that of an enzyme‐linked immunosorbent assay (ELISA) (1.11 ng/mL). The detection range was 0.66‐40 ng/mL. The intra‐assay coefficient of variation (CV) for sTim3 was 1.64%‐4.68%, and the inter‐assay CV was 5.72%‐9.32%. The cross‐reactivity to interleukin 6 (IL‐6) and kidney injury molecule 1 (KIM‐1) was 0.25% and 0.04%, respectively. The average recovery was 105.26%. The sTim3 concentration in patients with MN was considerably higher than that in healthy individuals (*P* < .001). The sTim3 concentration in the serum of patients with MN was significantly increased from G1 to G4 based on the Jonckheere‐Terpstra test (*P* < .001). Thus, we used sTim3 as a diagnostic indicator for distinguishing between healthy individuals and patients with MN as well as between different stages of MN.

**Conclusion:**

We successfully established TRFIA to detect sTim3 in serum. We then applied this method to patients with MN, demonstrating for the first time that TRFIA is a valid diagnostic tool to detect sTim3 in serum.

## 1 INTRODUCTION

T‐cell immunoglobulin and mucin domain 3 (Tim3) is a type 1 transmembrane receptor that can inhibit the activation of T helper 1 (Th1) cells after ligand binding.^1^ As a member of the Tim family, Tim3 was originally discovered on activated Th1 and Th17 cells, as well as T cell 1 (Tc1), where it induced T‐cell exhaustion or death after binding to galectin‐9 (gal‐9),^2^ partly through the T‐cell receptor (TCR) signaling pathway.^3^ Tim3 also plays an important role in adjusting the activity of monocytes, endothelial cells, macrophages, mast cells,^4^ natural killer cells, and dendritic cells. Though the overall mechanisms are still unclear, an imbalance of the Tim3 expression in innate immune cells may result in excessive or inhibited inflammatory reactions, which can contribute to an autoimmune response or evasion of viruses or tumor cells.^2^

Tim3 has a soluble form (sTim3) and a membrane‐bound form. sTim3 can be obtained by shearing with the help of enzymes.^5^ Möller‐Hackbarth et al^6^ identified a disintegrin and metalloprotease 10 (ADAM10) and ADAM17 as major sheddases of Tim3. It was also confirmed that another source of sTim3 is spleen cells. sTim3 mRNA is only found in spleen cells, and the activation of spleen cells leads to upregulation of sTim3.^7^ Xiao et al^8^ found that sTim3 and soluble programmed cell death protein 1 (sPD‐1) can enhance the cell‐mediated immune response induced by the simian immunodeficiency virus (SIV) vaccine in mice. High expression of membrane‐bound Tim3 was observed in common autoimmune diseases, like IgA nephropathy^9^ and systemic lupus erythematosus. These studies demonstrate that Tim3 is involved in pathogenesis; thus, Tim3 may be a potential therapeutic target for these diseases.^10^ Membranous nephropathy (MN) is a serious autoimmune disease and one of the main causes of adult nephrotic syndrome, which can lead to end‐stage renal failure.^11^ In addition, the role of sTim3 in MN has not been thoroughly studied.

In previous research, an enzyme‐linked immunosorbent assay (ELISA) was used to detect sTim3 in patient serum.^5^, ^12^ No other detection methods for sTim3 have been reported except for ELISA. Because ELISA uses enzymes to label the antibody or antigen, it greatly affects the biological activity of the labeled compounds. In addition, many false‐positive results are obtained due to the low sensitivity of ELISA. Insufficient stability and detection range are also common disadvantages of this method.^13^ Time‐resolved fluorescence immunoassay (TRFIA) is an alternative and non‐isotope immunoassay that uses lanthanide elements to label antigens or antibodies. It has the advantages of high sensitivity, good stability, and wide detection range compared with other detection methods. Therefore, this successful method of detection is widely used in the biomedical field.^14^, ^15^, ^16^ In this research, we aimed to establish a valid method to detect sTim3 and to evaluate clinical value of the method in detecting sTim3 in MN.

## 2 MATERIALS AND METHODS

### 2.1 Reagents and instruments

Two monoclonal antibodies against different epitopes of Tim3, capture (10390‐R024, Rabbit MAb) and detection antibody (10390‐MM04, Mouse MAb), along with Tim3 standard and Human Tim3 ELISA Kit, were purchased from Sino Biological Inc. Tris and bovine serum albumin (BSA) were obtained from Guangzhou Saiguo Biotech Co., Ltd. Standard buffer, assay buffer, enhancement solution, blocking buffer, and other reagents were supplied by Jiangsu Key Laboratory of Molecular Nuclear Medicine. A Sephadex‐G50 column was purchased from Seebio Biotech Co., Ltd., and Ultracel‐50K ultrafiltration tubes were purchased from Millipore. Ninety‐six–well plates were purchased from Xiamen Yunpeng Technology Development Co., Ltd. A micro‐oscillator was purchased from Jiangsu Kangjian Medical Products Co., Ltd. The DEM‐3 plate washer was purchased from Guangzhou Darui Biotechnology Co., Ltd. A time‐resolved immunofluorescence analyzer was purchased from Foshan Daan Medical Equipment Co., Ltd. An electrothermal constant temperature incubator was purchased from Shanghai Jinghong Laboratory Instrument Co., Ltd.

### 2.2 Buffer composition

The buffer solutions used in the assay are as follows: coating buffer (50 mmol/L Na_2_CO_3_‐NaHCO_3_, pH 9.6), labeling buffer (50 mmol/L Na_2_CO_3_‐NaHCO_3_, pH 9.0), and elution buffer (50 mmol/L Tris‐HCl, containing 0.2% BSA, 0.05% Proclin, pH 7.8). The compositions of the standard buffer, assay buffer, blocking buffer, enhancement solution, washing buffer, and other buffers have been previously reported.^17^

### 2.3 Serum samples

The sera of 47 healthy individuals and 54 patients with MN were obtained from Wuxi People's Hospital affiliated to Nanjing Medical University. Blood samples were centrifuged at 1200 *g*for 5 minutes to prepare the serum, and the serum samples were stored at −20°C until use. The serum samples did not undergo hemolysis or lipemia during the preparation process. The inclusion criteria for healthy subjects were that they did not have a history of kidney disease, autoimmune disorders, or infections with viruses such as the human immunodeficiency virus, hepatitis B virus, and hepatitis C virus. All registered subjects provided written, informed consent to participate in this research. The research program was approved by Wuxi People's Hospital affiliated to Nanjing Medical University.

### 2.4 Coating of the microwell plates

The original concentration of the Tim3 antibody (Tim3‐R024, capture antibody) was 1 mg/mL. First, it was diluted with coating buffer to 3 μg/mL. Then, 100 μL of 3 μg/mL Tim3 antibody was added into each well of a 96‐well microtiter plate, and the plate was sealed and incubated overnight at 4°C. Next, the plate was washed once, after which each well was blocked with 150 μL of blocking buffer containing 3 g/L BSA at room temperature for 2 hours. Finally, the blocking buffer was decanted, and the plates were dried under vacuum, followed by storage in a sealed plastic bag with a desiccant at −20°C.

### 2.5 Antibody labeling

Tim3 antibody labeling was conducted based on the manufacturer's instructions. First, 300 μL of the Tim3 antibody (Tim3‐MM04, detection antibody) was transferred to an ultrafiltration tube and centrifuged at 7100 *g* for 8 minutes. Then, 200 μL of labeling buffer was added, and the tube was centrifuged at 7100 *g* for 8 minutes; this step was repeated seven times. Next, 50 μL of labeling buffer was added to the ultrafiltration tube, and the tube was centrifuged at 1000 *g* for 1 minute; this step was repeated twice. The antibody was mixed with the 60 μg of diethylenetriaminetriacetic acid (DTTA)‐Eu^3+^ and incubated overnight at 30°C with shaking. The labeled antibody was purified by Sephadex‐G50, and the purified labeled antibody was stored in elution buffer containing 0.2% BSA. Finally, the antibody was collected and stored at −20°C.

### 2.6 Assay protocol

Tim3 standards (100 ng/mL) were diluted with standard buffer containing 0.5% BSA to final concentrations of 0, 5, 10, 20, and 40 ng/mL. Then, TRFIA of sTim3 was conducted using a two‐step, noncompetitive “sandwich‐type” technique. A two‐step method was used because the serum contained EDTA. First, 20 μL each of the standard and serum samples was added into a 96‐well microtiter plate. Next, 80 μL of assay buffer was added into each well of the coated microtiter plate. The plate was incubated at 37°C with gentle shaking for 1 hour. After the incubation, the plate was washed twice with washing buffer, and 100 μL of 200‐fold diluted Eu^3+^‐labeled Tim3‐MM04 solution was then added. Finally, the plate was incubated again at 37°C with gentle shaking for 2 hours. After six washes, 100 μL of enhancement solution was added. The plate was gently shaken for 3 minutes, and the fluorescence was then measured. The sTim3 concentration of the serum sample was calculated based on the Tim3 standard curve.

### 2.7 Assessment of the sTim3 TRFIA method

#### 2.7.1 Sensitivity and linearity

The standard curve of Tim3 consisted of different concentrations of standards (0, 5, 10, 20, and 40 ng/mL). Linear regression analysis was performed, and the equation and the coefficient of determination, *R* ^2^, are shown as indicators of linearity. The background fluorescence intensity of 10 replicates of the 0 ng/mL standard was measured, and the sensitivity was calculated as the mean + 2SD.^18^

#### 2.7.2 Precision

The precision of the method was assessed using three serum samples from patients with MN that measured low, medium, and high sTim3 concentrations. To obtain intra‐assay and inter‐assay precision, 10 independent experiments were conducted for each of the three samples.

#### 2.7.3 Specificity

The specificity was assessed using kidney injury molecule‐1 (KIM‐1) and interleukin 6 (IL‐6) as potential interfering antigens. The presence of cross‐reactions was determined by detecting the final fluorescence value in the presence of the antigen. Ten independent experiments were performed for each antigen.

#### 2.7.4 Recovery

The recovery was assessed by adding 100 ng/mL Tim3 standards to three serum samples with known concentrations of sTim3 at a ratio of 1:9. The original concentrations of the three serum samples were 11.8, 25.4, and 61.2 ng/mL, respectively. After the Tim3 standards had been added, the final theoretical concentrations of sTim3 in the three samples were 20.62, 32.86, and 65.08 ng/mL, respectively. The following equation was used: recovery (%) = (measured concentrating/theoretical concentration) × 100%.

#### 2.7.5 Method comparison

To assess the correlation between TRFIA and ELISA, the sTim3 concentrations of 36 clinical serum samples were determined using both a commercial kit (ELISA) and the newly established TRFIA. The ELISA experiment was performed according to the manufacturer's instructions.

### 2.8 Clinical application of TRFIA

To demonstrate the preliminary application of TRFIA, we selected patients with MN as study subjects and evaluated the clinical value of the detection method for sTim3 in these patients. According to the internationally recognized staging criteria for glomerular filtration rate (GFR), we divided all patients with MN into four stages: G1 (≥90 mL/min/1.73 m^2^), G2 (60‐89 mL/min/1.73 m^2^), G3 (30‐59 mL/min/1.73 m^2^), and G4 (15‐29 mL/min/1.73 m^2^). We measured the sTim3 concentration in the serum by TRFIA; other patient parameters had been previously determined at the Wuxi People's Hospital affiliated to Nanjing Medical University.

### 2.9 Statistical analysis

The data were expressed as the mean ± SD. SPSS16.0 (Chicago, IL, USA) was used to analyze the data, and Prism 7.00 (San Diego, CA, USA) was used to prepare the graphs. Spearman correlation coefficients were used to assess whether the sTim3 concentration correlated with various parameters. sTim3 concentrations and various clinical parameters at different GFRs were analyzed using the Jonckheere‐Terpstra test. *P* values <.05 were considered statistically significant.

## 3 RESULTS

### 3.1 Assay evaluation

**Figure 1:**
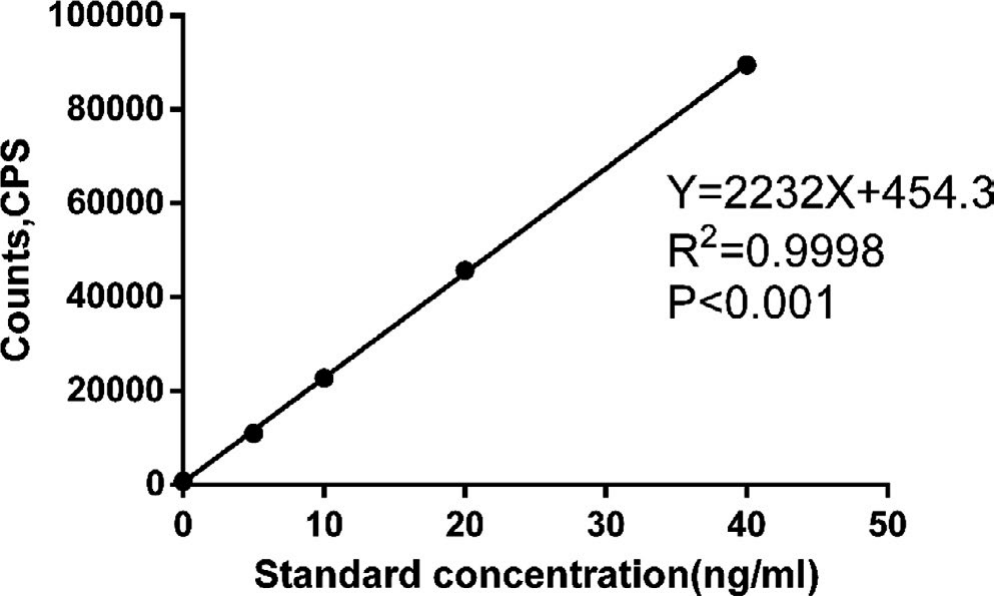
Standard curve of Tim3 (*P* < .001). CPS (counts per second)

The sensitivity and linearity results are presented in Figure 1. The detection range for sTim3 was 0.66‐40 ng/mL. The *R* ^2^ of the Tim3 standard curve was .9998, indicating a good linear relationship between the sTim3 concentration and the fluorescence intensity. The sensitivity of TRFIA was 0.66 ng/mL, which was higher than that of ELISA (1.11 ng/mL). The precision was measured by the coefficient of variation (CV). The intra‐assay CV for sTim3 varied between 1.64% and 4.68%, and the inter‐assay CV varied between 5.72% and 9.32% (Table 1). All CV values were less than 10%, suggesting that the precision of the experiment was acceptable. Table 2 shows that the cross‐reactivities of IL‐6 and KIM‐1 were 0.25% and 0.04%, respectively. The extremely low cross‐reactivity observed showed that TRFIA had high specificity for sTim3. As shown in Table 3, the average recovery was 105.26%. The results showed that the experiment was free from interferences in serum.

**Table 1:** Precision of the time‐resolved fluorescence immunoassay

	Concentration	Average (ng/ml)	Standard deviation	CV (%)
Inter‐assay (n = 10)	Low	6.95	0.62	8.95
	Medium	22.52	1.29	5.72
	High	51.68	4.81	9.32
Intra‐assay (n = 10)	Low	7.27	0.34	4.68
	Medium	21.95	0.67	3.05
	High	49.30	0.81	1.64

**Table 2:** Cross‐reactivities of the time‐resolved fluorescence immunoassay (n = 10)

Interferent (ng/ml)	Concentration (ng/ml)	Determined (ng/ml)	Cross‐reactivity (%)
Tim3	20.00	20.32	101.62
IL‐6	200.00	0.49	0.25
KIM‐1	1000.00	0.43	0.04

**Table 3:** Recoveries of the time‐resolved fluorescence immunoassay (n = 3)

Samples	Theoretical concentration (ng/ml）	Measured (ng/ml）	Recovery (%)
1	20.62	22.02	106.79
	32.86	36.23	110.26
	65.08	65.03	99.92
2	20.62	23.62	114.55
	32.86	38.03	115.73
	65.08	60.65	93.19
3	20.62	20.03	97.14
	32.86	34.67	105.51
	65.08	67.82	104.21
Average recovery (%) 105.26			

### 3.2 Correlation with ELISA

**Figure 2:**
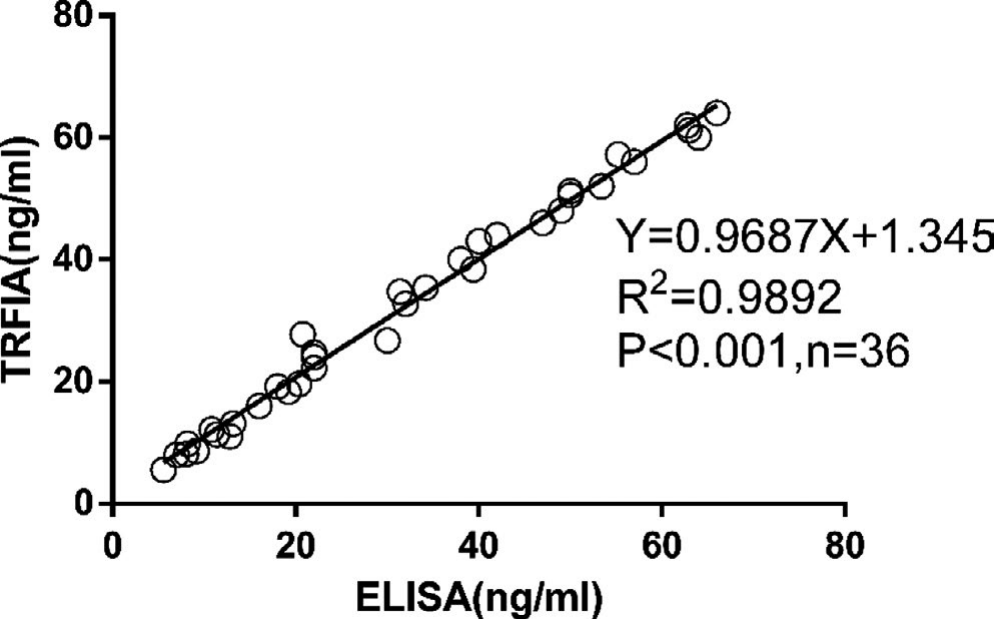
Correlation of sTim3 concentration results between ELISA and the established time‐resolved fluorescence immunoassay (TRFIA)

Thirty‐six clinical serum samples were analyzed by ELISA and the newly established TRFIA (Figure 2). The sTim3 concentrations detected by the two methods were highly similar, as indicated by the linear fit with the equation y = 0.9687x + 1.345 and *R* ^2^ of .9892 (*P* < .001). The results demonstrated that TRFIA is a reliable technique for the detection of sTim3 in clinical serum samples.

### 3.3 Clinical application

**Figure 3:**
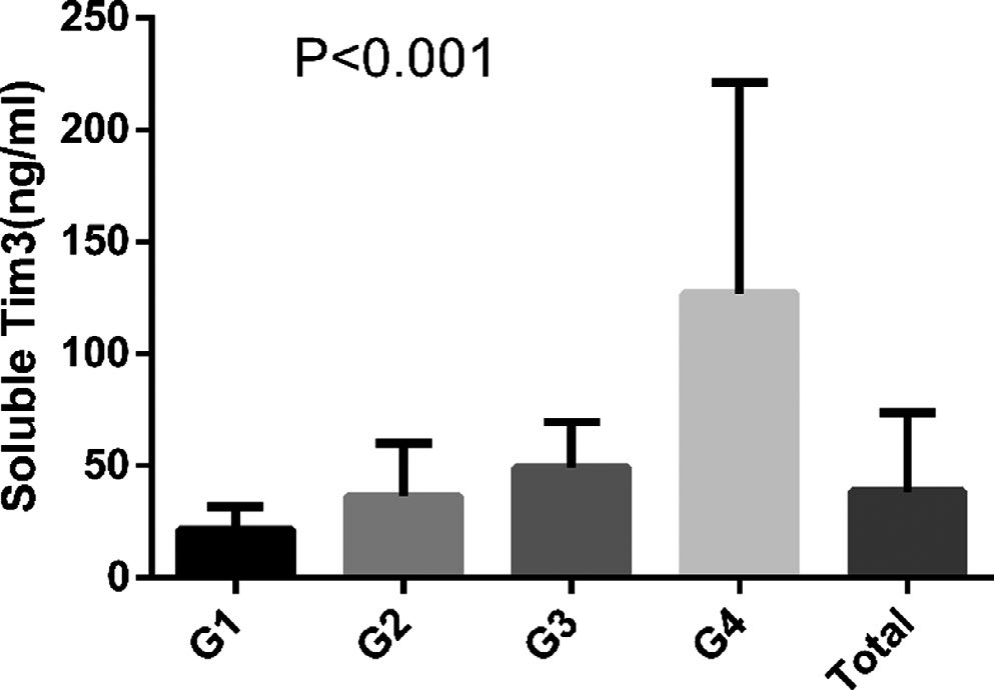
sTim3 concentration in different GFR stages of patients with MN (*P* < .001), Jonckheere‐Terpstra test was used to analyze *P* value, and *P* < .05 was considered significant

We investigated the sTim3 concentration in various stages of MN. Age, systolic blood pressure, urea, creatinine, urinary protein, and osmotic pressure were noticeably increased from G1 to G4, based on the Jonckheere‐Terpstra test (Table 4). The sTim3 concentration in the serum of patients with MN was also significantly increased from G1 to G4 based on the Jonckheere‐Terpstra test (*P* < .001, Figure 3 and Table 4).

**Table 4:** Comparison of various parameters in different GFR stages of MN (n = 54)

Parameters	G1	G2	G3	G4	Total	Jonckheere‐Terpstra
Number (male/female)	20 (8/12)	20 (12/8)	11 (11/0)	3 (2/1)	54 (33/21)	0.004
Age (y)	44.8 ± 10.7	59.9 ± 11.0	64.5 ± 2.1	53 ± 8.5	54.9 ± 12.6	1.89 × 10^‐4^
SBP (mm Hg)	120.4 ± 12.0	127.6 ± 20.3	132.0 ± 13.6	134.0 ± 8.5	126.1 ± 16.5	0.033
DBP (mm Hg)	76.2 ± 9.5	79.4 ± 11.5	82.0 ± 9.8	86.0 ± 8.6	79.1 ± 10.7	0.095
ALB (g/L)	28.3 ± 5.8	26.9 ± 7.3	24.1 ± 8.9	19.6 ± 6.8	26.4 ± 7.5	0.052
UREA (mmol/L)	4.3 ± 1.3	7.1 ± 2.0	11.0 ± 3.7	9.1 ± 2.0	7.0 ± 3.4	1.34 × 10^‐9^
CREA (μmol/L)	58.4 ± 12.5	87.4 ± 18.0	138.4 ± 17.9	253.2 ± 61.1	96.2 ± 52.3	2.32 × 10^‐13^
U‐PRO (g/L)	1.6 ± 1.3	2.1 ± 2.1	3.3 ± 2.0	4.75 ± 0.8	2.3 ± 2.0	0.012
eGFR (mL/min/1.73 m^2^)	110.5 ± 10.4	75.6 ± 10.0	47.6 ± 7.4	23.2 ± 5.4	79.9 ± 28.9	3.38 × 10^‐15^
URIC (μmol/L)	366.1 ± 58.1	400.8 ± 91.6	431.4 ± 91.0	199.3 ± 54.3	383.0 ± 93.7	0.461
OSM‐B (mmol/kg)	280.6 ± 4.2	285 ± 3.8	286.1 ± 3.7	279.7 ± 5.0	283.3 ± 4.7	0.008
Pla2r‐Ab (RU/mL)	60.9 ± 75.6	151.5 ± 363.3	77.8 ± 109.5	21.1 ± 26.9	95.7 ± 235.5	0.680
sTim3 (ng/mL)	21.4 ± 10.2	36.4 ± 23.1	49.2 ± 19.3	126.9 ± 77.0	38.5 ± 34.8	6.88 × 10^‐6^

Note

Data were presented as mean ± SD. sTim3 concentration and various clinical parameters in different GFR stages were analyzed by Jonckheere‐Terpstra test. *P* < .05 was considered significant.

Abbreviations: ALB, albumin; CREA, serum creatinine; DBP, diastolic blood pressure; eGFR, estimated glomerular filtration rate; OSM‐B, osmotic pressure; Pla2r‐Ab, pla2r‐antibody; SBP, systolic blood pressure; sTim3, soluble Tim3; U‐PRO, urinary protein; UREA, urea; URIC, uric acid.

**Figure 4:**
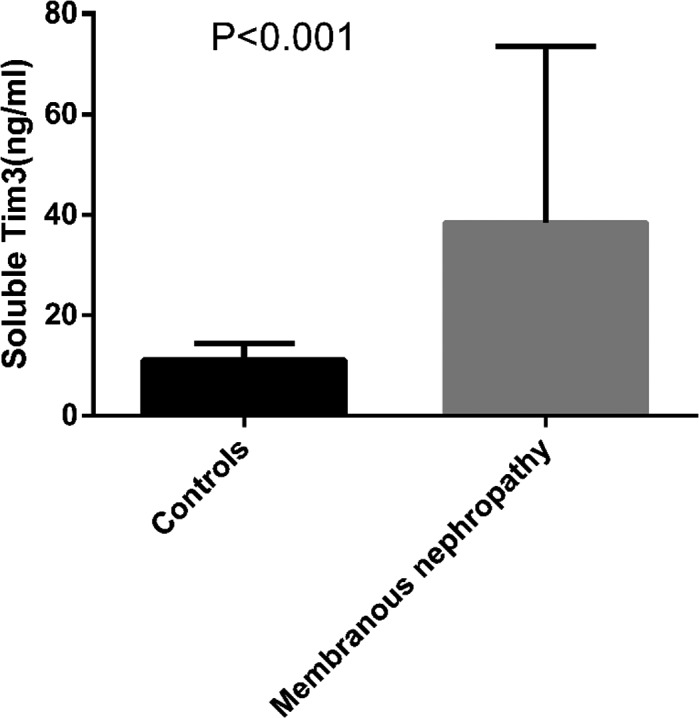
sTim3 concentration in patients with MN (n = 54) and normal control (n = 47, *P* < .001), *t* test was used to analyze *P* value, *P* < .05 was considered significant

**Figure 5:**
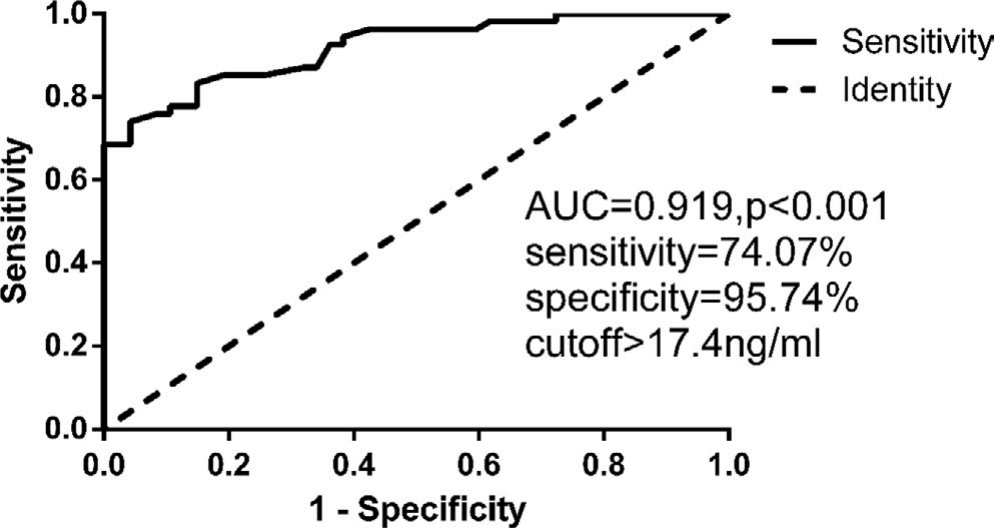
ROC analysis on sTim3 in order to distinguish MN from normal control (*P* < .001). AUC, area under ROC curve; and ROC, receiver operating characteristic

**Figure 6:**
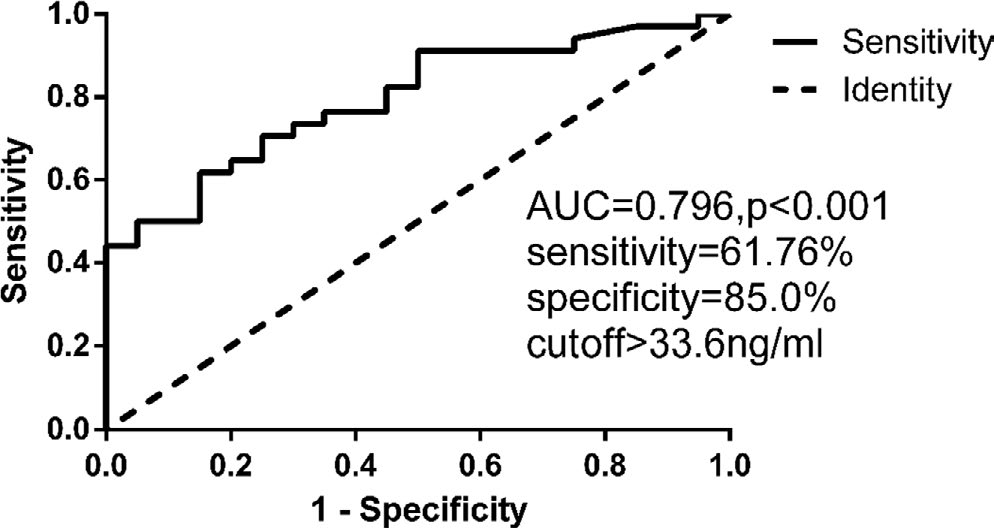
ROC for the sTim3 which distinguished G1 from G2, G3 and G4 (*P* < .001)

As shown in Figure 4, the sTim3 concentration in patients was 38.5 ± 34.8 ng/mL (n = 54), which was significantly higher than that in healthy people (11.1 ± 3.3 ng/mL, n = 47, *P* < .001). Receiver operating characteristic (ROC) analysis of the sTim3 concentration in patients with MN is presented in Figure 5 and Figure 6. As shown in the ROC curve, sTim3 can be used as a reliable indicator to distinguish patients with MN from healthy controls (area under the curve, AUC = 0.919), with a cutoff value of 17.4 ng/mL. The sensitivity was 74.07%, and the specificity was 95.74% (Figure 5). At the same time, this indicator can also be used to distinguish G1 from other stages of MN (AUC = 0.796), with a cutoff value of 33.6 ng/mL. The sensitivity was 61.76%, and the specificity was 85.0% (Figure 6). The above results indicated that sTim3 can be used as a diagnostic indicator for distinguishing between healthy individuals and patients with MN as well as between different stages of MN.

Our observation from the correlation analysis between the sTim3 concentration and other parameters resulted in additional findings (Table 5). The sTim3 concentration was positively correlated with age (*r* = .398, *P* = .003), systolic blood pressure (*r* = .395, *P* = .003), urea (*r* = .444, *P* < .001), serum creatinine (*r* = .509, *P* < .001), urinary protein (*r* = .718, *P* < .001), and phospholipase A2 receptor antibody (Pla2r‐Ab, *r* = .370, *P* = .006). In contrast, the sTim3 concentration was negatively correlated with albumin (*r* = −.672, *P* < .001) and glomerular filtration rate (*r* = −.587, *P* < .001). There was no correlation between the sTim3 concentration and diastolic blood pressure (*P* = .195), osmotic pressure (*P* = .997), or uric acid level (*P* = .624).

**Table 5:** Correlation of sTim3 level with other parameters

Parameters	Correlation coefficient (*r*)	*P* value
Age	.398	.003
SBP	.395	.003
DBP	.179	.195
ALB	−.672	<.001
UREA	.444	<.001
CREA	.509	<.001
U‐PRO	.718	<.001
eGFR	−.587	<.001
URIC	.068	.624
OSM‐B	.97 × 10^‐4^	.997
Pla2r‐Ab	.370	.006

Note

Correlation of sTim3 concentration with other parameters was determined by spearman correlation analysis. *P* < .05 was considered significant.

## 4 DISCUSSION

Based on the existing literature, this is the first study of TRFIA used for the detection of sTim3 and its application to test sera from patients with MN. This study revealed that sTim3, as an immune system‐related protein, reflected not only the immune response state of patients with MN but also the severity of their renal function. Thus, sTim3 in serum can be a diagnostic indicator of MN. In some preclinical tumor models, blocking of Tim3, along with other checkpoint inhibitors, enhances the antitumor immune response, which can inhibit tumor growth.^19^ This result suggests that Tim3 may be a potential therapeutic target for MN. At present, the research on Tim3 is focused on membrane‐bound Tim3; sTim3 is detached from the cell membrane. Therefore, the sTim3 concentration may reflect the expression level of membrane‐bound Tim3.^5^ It is known that interactions between Tim3 and its ligand gal‐9 play an important regulatory role in transplantation,^1^, ^20^ chronic infection,^21^, ^22^, ^23^ autoimmune disorders,^10^, ^24^, ^25^ and tumor immunity.^5^, ^26^, ^27^, ^28^, ^29^, ^30^, ^31^, ^32^, ^33^, ^34^ However, the roles of Tim3 and sTim3 in pathogenesis have not been fully revealed.

Currently, commercial kits for detecting sTim3 are limited to the ELISA format. Although ELISAs are very popular detection methods in the biomedical field, the ELISA platform has many disadvantages. For example, enzyme‐labeled compounds are unstable, and labeled enzymes are susceptible to inactivation by the environment.^35^ In addition, the labeling process is complicated and requires highly skilled personnel. Furthermore, the detection process is complex and time‐consuming. Compared with ELISA, TRFIA has minimal effect on biological activity because of its atomic labeling. Moreover, TRFIA has higher sensitivity than other methods, with a detection limit of 10^−18^ mol/L, which is markedly improved compared with that of ELISA (10^−9^ mol/L) and chemiluminescence (10^−15^ mol/L).^13^ In the present study, TRFIA also showed higher sensitivity than ELISA (0.66 ng/mL vs 1.11 ng/mL). Further, the detection procedure of TRFIA is simple and fast. The ELISA requires more complex steps after secondary antibody binding, which include the addition of substrate solution, incubation for 20 minutes at room temperature, followed by addition of stop solution; on the contrary, TRFIA only requires addition of enhancement solution and shaking for 3 minutes. Besides, the step of labeling the antibodies with lanthanides is easy because rare earth ions react readily with proteins. These advantages of TRFIA over ELISA are expected to promote its adoption in clinical applications. Thus, the developed TRFIA is a convenient, highly sensitive, and inexpensive method for the detection of sTim3. In addition to sTim3, there are many other immune checkpoint proteins, such as programmed cell death protein 1 (PD‐1) and cytotoxic T lymphocyte–associated antigen 4 (CTLA‐4), which also have soluble forms. Highly quantitative and sensitive detection of the concentration of immunological checkpoint proteins in serum is important for the diagnosis and treatment of many diseases such as tumors and viral infections. Therefore, we predict that TRFIA will be applied to the detection of additional immune checkpoint proteins in serum.

In conclusion, we successfully established TRFIA for the detection of sTim3. We also found that the sTim3 concentration was significantly correlated with renal function indicators in patients with MN, which can distinguish patients with MN and help better diagnosis.
